# Dynamic Control of Phase Matching in Nonlinear Metasurfaces Using Pancharatnam–Berry Phase

**DOI:** 10.1002/nap2.70273

**Published:** 2026-08-28

**Authors:** Madona Mekhael, Roman Calpe, Tommi K. Hakala, Robert Fickler, Mikko J. Huttunen

**Affiliations:** ^1^ Photonics Laboratory Physics Unit Tampere University Tampere Finland; ^2^ Center for Photonics Sciences University of Eastern Finland Joensuu Finland

**Keywords:** multipass cell, nonlinear metasurfaces, Pancharatnam–Berry phase, phase matching, plasmonic nanostructures, reconfigurable nonlinear optics, second‐harmonic generation

## Abstract

Reconfiguring the spectral output of nonlinear metasurfaces after fabrication remains challenging. We address this by exploiting the nonlinear Pancharatnam–Berry phase of $C_{3v}$‐symmetric plasmonic metasurfaces. By integrating two metasurfaces inside a multipass cell, we experimentally demonstrate continuous spectral tuning of second‐harmonic generation (SHG) phase‐matching peaks across a 900–970 nm pump range by rotating one metasurface relative to the other. The extracted geometric phase follows the 3σθ dependence, and a full 2π tuning cycle is completed with 120° of physical rotation. This establishes geometric‐phase metasurfaces as a reconfigurable nonlinear platform, where mechanical rotation enables post‐fabrication and broadband tuning of nonlinear optical responses.

## Graphical abstract

Can mechanical rotation of a metasurface control the phase of a nonlinear optical signal? In a dual‐metasurface multipass cell, a circularly polarized pump interacts repeatedly with two plasmonic metasurfaces between two mirrors. By exploiting the geometric phase, rotating one metasurface shifts the phase‐matching condition of the second‐harmonic signal across its full range, enabling continuous and reversible spectral tuning of the nonlinear output.

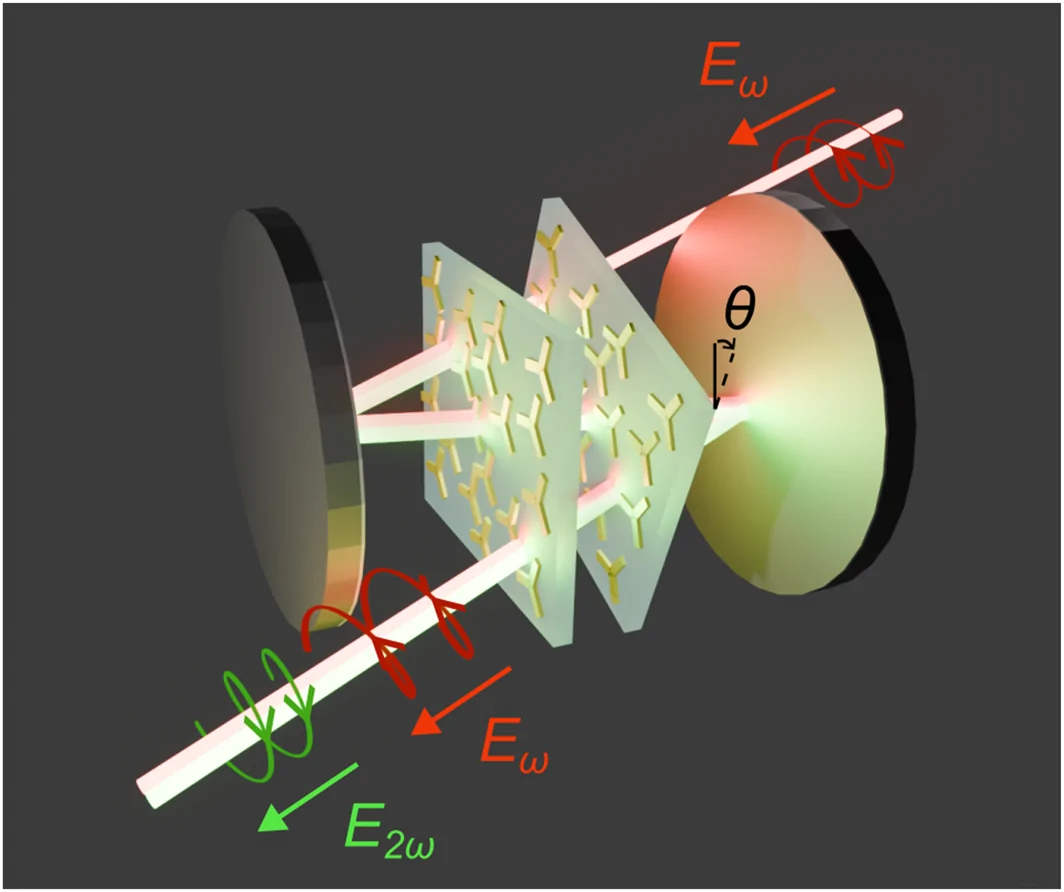

## 1 Introduction

The ability to tailor nonlinear optical responses at the sub‐wavelength scale has emerged as a cornerstone of modern nanophotonics, offering a flat alternative to traditional bulk nonlinear crystals. Unlike bulk media, which require long interaction lengths for efficient nonlinear processes [1, 2], optical metasurfaces enable nonlinear phenomena within a sub‐wavelength thickness by enhancing light–matter interactions through engineered resonances, resulting in relaxed phase‐matching requirements [3, 4, 5, 6, 7, 8, 9, 10, 11, 12]. A defining advantage of metasurfaces is their ability to exert precise control over the local phase of an optical signal, enabling transformative applications in the linear regime such as flat lensing [13, 14, 15], holography [16, 17, 18, 19], and arbitrary beam shaping [20, 21, 22]. Nonlinear metasurfaces extend these capabilities into the frequency‐conversion regime, enabling sophisticated wavefront engineering of the generated signal [23, 24, 25, 26]. Among the various mechanisms for phase control, the Pancharatnam–Berry (PB) phase—also known as the geometric phase—has proven exceptionally robust. Because the PB phase is determined entirely by the spatial orientation of the meta‐atoms, it provides a deterministic and broadband method for phase manipulation [27, 28, 29]. While the nonlinear PB phase has been exploited for spatial wavefront control such as holography and beam steering [23, 30, 31], its potential for dynamic tuning of nonlinear phase‐matching conditions has remained unexplored.

More broadly, the vast majority of nonlinear metasurfaces are static: once fabricated, their phase profiles and resulting emission characteristics are fixed. This imposes a fundamental constraint on practical applications—for instance, a nonlinear metasurface designed for a specific phase‐matching wavelength cannot be re‐tuned to a different operation wavelength without fabricating an entirely new device. The ability to reconfigure the nonlinear response after fabrication would therefore be highly valuable, enabling wavelength‐agile nonlinear sources and adaptive nonlinear optical platforms that can be optimized post‐fabrication without the need for repeated nanofabrication. Achieving dynamic tunability—the ability to reconfigure the nonlinear response in real time—remains a significant challenge in the field. Existing approaches mainly rely on external stimuli such as thermal heating [32] or electrical gating [33, 34, 35, 36, 37, 38]. Thermal approaches are inherently slow due to the timescales associated with transfer of thermal energy. Approaches based on electrical gating, although faster, require specific materials (such as ferro‐electric lithium niobate), complex device integration and electrical contacts. Both approaches typically provide limited tuning ranges, as they rely on material‐dependent effects—such as the thermo‐optic or electro‐optic effect—which produce only small refractive index changes. Furthermore, above methods are volatile methods, requiring continuous energy input to maintain the device in the tuned state [39]. An alternative approach that has remained unexplored is to exploit the inherent rotational sensitivity of the PB phase: mechanically adjusting the relative orientation between nonlinear elements enables continuous phase modulation without altering the physical properties of the nanostructures. Mechanical rotation requires no electrical contacts or active materials, is inherently broadband and non‐volatile method, and provides a complete 2π phase tuning range by geometric means alone. We acknowledge that mechanical actuation is slower than electrical switching. However, compact rotation stages and micro‐electromechanical systems (MEMS) actuators offer a practical path toward miniaturized and faster implementations.

In this work, we demonstrate a dynamic approach for nonlinear wavefront control by integrating a dual‐metasurface configuration within a multipass cell (MPC), schematically presented in Figure 1a. One metasurface serves as a stationary reference while a second, mounted on a rotation stage, acts as a tunable phase actuator. Cascaded interactions between the pump beam and both metasurfaces generate phase‐matching peaks in the SHG spectra, whose spectral positions are governed by the metasurface geometry. Unlike our previous demonstration of static phase‐matching peaks in a single‐metasurface MPC [40], where the peak positions were fixed by the metasurface design, the present dual‐metasurface configuration introduces mechanical rotation as a freely adjustable degree of freedom for continuous spectral tuning. By rotating one metasurface with respect to the other, we exploit the nonlinear geometric phase to introduce a continuously controllable phase delay of up to 2π into the conversion process, enabling deterministic spectral translation of the SHG phase‐matching peaks. This platform offers a versatile and compact route toward reconfigurable nonlinear flat‐optics, with potential applications in tunable frequency conversion, nonlinear holography, and on‐chip optical signal processing.

**FIGURE 1:**
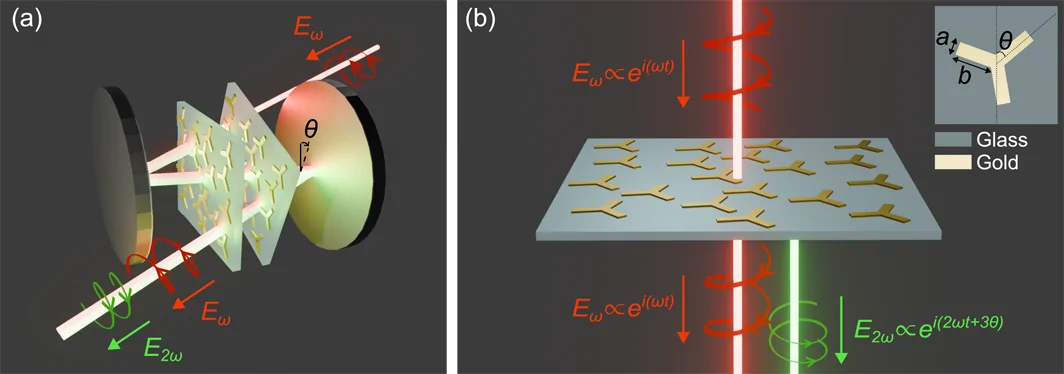
Geometric phase control in a multipass cell. (a) Schematic of the dual‐metasurface multipass cell, consisting of two high‐reflectivity mirrors enclosing two metasurfaces, one of which is rotated by an angle θ relative to the other. (b) Schematic of the metasurface illuminated by a right‐circularly polarized (RCP) fundamental pump at frequency $E_{\omega }$. While the transmitted pump remains unmodulated, the generated second harmonic $E_{2\omega }$ is emitted in the left‐circular polarization (LCP) state and acquires a nonlinear PB phase determined by the in‐plane orientation angle θ of the meta‐atoms. The inset shows a single three‐bladed gold nanostructure with arm width a=50 nm and arm length b=130 nm.

## 2 Nonlinear Phase‐Matching via the Geometric Phase

In the MPC (Figure 1a), the pump interacts repeatedly with the metasurfaces, making the SHG analogous to that of stacked metasurfaces [41]. On each pass, the pump generates a SHG signal that interferes constructively or destructively with the SHG from previous passes, depending on the accumulated phase mismatch between the fundamental and second‐harmonic fields. The total SHG intensity is given by:

(1) $$\text{SHG}\propto {\left|\sum_{j=1}^{N}T{\left(\omega \right)}^{j}T{\left(2\omega \right)}^{j/2}e^{ij\Delta k}\chi_{\text{ms}}^{\left(2\right)}E{\left(\omega \right)}^{2}\right|}^{2},$$

where $\chi_{\text{ms}}^{\left(2\right)}$ is the relevant component of the nonlinear susceptibility tensor of the metasurface, N is the number of passes, T(ω) and T(2ω) are the transmittances at the fundamental and second‐harmonic frequencies, and Δk is the phase mismatch. When the pump wavelength is scanned, the interference of the SHG signals gives rise to discrete peaks in the SHG spectrum at the phase‐matched wavelengths, whose spectral positions are determined by the metasurface design. In the static case, Δk depends solely on the propagation phase and the metasurface interaction phase at both the pump and SHG frequencies [40].

In the present work, we extend earlier framework by introducing the nonlinear PB phase as an additional tunable control parameter. The PB phase arises when a meta‐atom is rotated by an angle θ: for a meta‐atom generating the nth harmonic under circularly polarized illumination, the emitted signal acquires a spin‐dependent geometric phase:

(2) $$\Phi_{\text{PB}}=\left(n\pm 1\right)\sigma \theta ,$$

where n is the harmonic order, σ=±1 is the helicity of the incident circular polarization (with σ=+1 for RCP and σ=−1 for LCP), and the + and − signs correspond to output light in the opposite (+, cross‐polarized) and same (−, co‐polarized) circular polarization state as the input, respectively [30]. The allowed harmonic orders are governed by the rotational symmetry of the meta‐atom [30, 42]. A meta‐atom with m‐fold rotational symmetry can only generate harmonics of order:

(3) n=jm±1,

where j is a positive integer, the sign is + for the co‐polarization and − for cross‐polarization. Adding this contribution, the full phase‐matching condition $\Delta k+\Phi_{\text{PB}}=2\pi l$ becomes:

(4) $$2\left(\varphi_{\omega }+\delta_{\omega }\right)-\varphi_{2\omega }-\delta_{2\omega }+\Phi_{\text{PB}}=2\pi l,$$

where l is an integer, $\varphi_{\omega }=k_{\omega }h$ and $\varphi_{2\omega }=k_{2\omega }h$ are the phase accumulations over a propagation distance h, with $k_{\omega }=2\pi /\lambda_{\omega }$ and $k_{2\omega }=4\pi /\lambda_{\omega }$ the wavenumbers in air, $\delta_{\omega }$ and $\delta_{2\omega }$ are the metasurface interaction phases extracted from transmittance spectra. The specific case of meta‐atoms with three‐fold rotational symmetry, as used in our experiment, are depicted in Figure 1b. Since $\Phi_{\text{PB}}$ depends solely on θ, it acts as a tunable bias that continuously shifts the SHG phase‐matching peaks without modifying the material or geometry of either metasurface.

## 3 Results and Discussion

### 3.1 Sample Design and Working Principle

The metasurface consists of three‐bladed gold nanoparticles with arm length of 130 nm and arm width of 50 nm, fabricated by electron‐beam lithography on a glass substrate. The structural parameters were determined through COMSOL Multiphysics simulations, with the primary criterion of ensuring the absence of plasmonic resonances within the 900–970 nm pump wavelength range. Operating away from resonance ensures that the phase‐matching condition is governed mainly by the geometric phase, without contributions from resonance‐induced phase shifts. At the same time, the parameters were chosen to maintain a sufficient nonlinear response for observable SHG signals. We note that the demonstrated tuning mechanism is not sensitive to the exact structural parameters. Any metasurface geometry generating SHG should exhibit the same geometric‐phase‐driven tuning behavior, depending on the rotational symmetry of the constituent nanoparticles, provided that no sharp resonances are present within the pump wavelength range. The meta‐atoms are distributed with a density of 6.5 particles/$\mu m^{2}$ in a randomized spatial arrangement, which suppresses collective lattice resonances [43, 44], while maintaining a strictly uniform global orientation θ. The fabricated nanostructures were characterized by scanning electron microscopy (Figure 2a,b), revealing arm lengths and widths in good agreement with the target geometry, with slight deviations attributable to fabrication tolerances. The measured transmittance spectra (Figure 2c) confirm the absence of plasmonic resonances in the 900–970 nm pump wavelength range.

**FIGURE 2:**
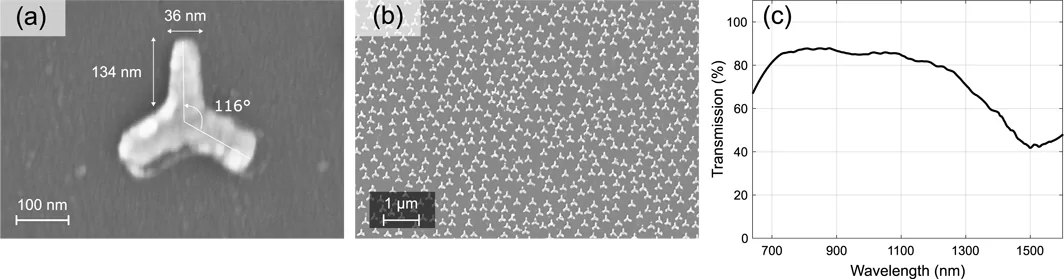
Sample characterization. (a) Scanning electron microscopy (SEM) image of a single fabricated three‐bladed gold nanostructure. The measured dimensions are indicated, showing slight deviations from the nominal design values due to fabrication tolerances. (b) SEM image of a larger metasurface area, demonstrating the high fabrication quality and uniformity of the nanostructure array. (c) Measured transmittance spectrum of the metasurface showing plasmon resonance around 1500 nm.

The three‐bladed geometry was chosen for its $C_{3v}$ rotational symmetry (m=3). From the selection rule of Equation (3), the lowest allowed harmonic order is n=jm−1=2 (with j=1), confirming that SHG is the dominant nonlinear process. Furthermore, the $C_{3v}$ selection rules permit only the cross‐polarized SHG channel, meaning the co‐polarized channel is forbidden. Applying Equation (2) with the + sign (cross‐polarized channel) and substituting n=2, the nonlinear PB phase imparted to the SHG signal is:

(5) $$\Phi_{\text{PB}}=3\sigma \theta ,$$

where σ=±1 is the helicity of the incident pump. Importantly, the transmitted fundamental pump acquires no geometric phase upon rotation. This means that rotating the metasurface modifies only the SHG phase, leaving the pump unaffected. Furthermore, the threefold amplification in Equation (5) means that a physical rotation of only 120° spans the full 2π phase range.

### 3.2 Experiments

The experiment was performed using a femtosecond Titanium:Sapphire laser (Coherent Chameleon Vision II) as the fundamental pump, scanned across a spectral range of 900–970 nm with a pulse duration of around 200 fs and a repetition rate of 80 MHz. To control the SHG phase via the nonlinear PB phase, the pump was converted to a right‐circularly polarized (RCP) state using a linear polarizer and a broadband zero‐order quarter‐wave plate. The average pump power incident on the first metasurface was maintained at 300 mW. The experimental setup is illustrated in Figure 3.

**FIGURE 3:**
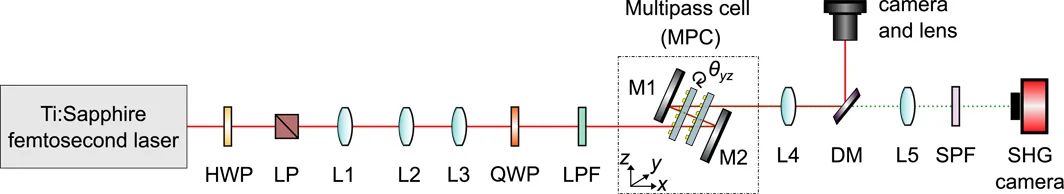
Experimental setup. Schematic of the setup used for SHG measurements from a dual‐metasurface multipass cell at different rotation angles of one metasurface. DM, dichroic mirror; HWP, half‐wave plate; L1–L3, beam‐shaping lenses; L4, L5, collection and focusing lenses; LP, linear polarizer; LPF, long‐pass filter; M1, M2, high‐reflectivity mirrors; QWP, quarter‐wave plate; SPF, short‐pass filter.

The MPC was constructed by placing two metasurface samples between two high‐reflectivity plane mirrors (R > 99%), separated by a distance of d = 3 cm. This separation was chosen to ensure that all interactions between the pump beam and both metasurfaces occur within the Rayleigh range of the beam (9.75 cm), guaranteeing an almost constant beam size across all passes. We note that a smaller separation would equally satisfy this condition without affecting the results. The mirrors were aligned to direct the beam through three passes per metasurface sample. This cascaded geometry establishes the interference‐driven phase‐matching condition responsible for the formation of discrete peaks in the SHG spectrum. Full details of the optical setup are provided in Supporting Information S1.

Both metasurfaces were initially co‐aligned at a global orientation of θ=0°. One metasurface remained fixed throughout, while the other was mounted on a motorized rotation stage and rotated in‐plane from 0° to 120° in steps of 15°. Owing to the threefold symmetry of the three‐bladed gold nanostructures, this 120° physical rotation spans a complete 2π cycle of the nonlinear geometric phase. The transmitted SHG signal was collected and analyzed via a sensitive CMOS camera (ZWO‐ASI1600MM).

As the geometric phase was tuned, the phase‐matching peaks underwent a continuous spectral translation across the 900–970 nm pump range as shown in Figure 4a. The angle‐resolved SHG map confirms this behavior, showing a diagonal shift of the intensity maxima with increasing rotation angle (Figure 4b). Upon reaching the full 120° rotation, each peak had shifted to the spectral position previously occupied by its neighbor, completing one full 2π geometric phase cycle. To quantify this, we extracted the geometric phase from sinusoidal fits to the SHG intensity at two representative wavelength regions, finding linear growth with rotation angle in good agreement with the ideal 3σθ dependence (Figure 4c). This deterministic and reversible spectral tuning demonstrates dynamic control over the phase‐matching condition of the SHG.

**FIGURE 4:**
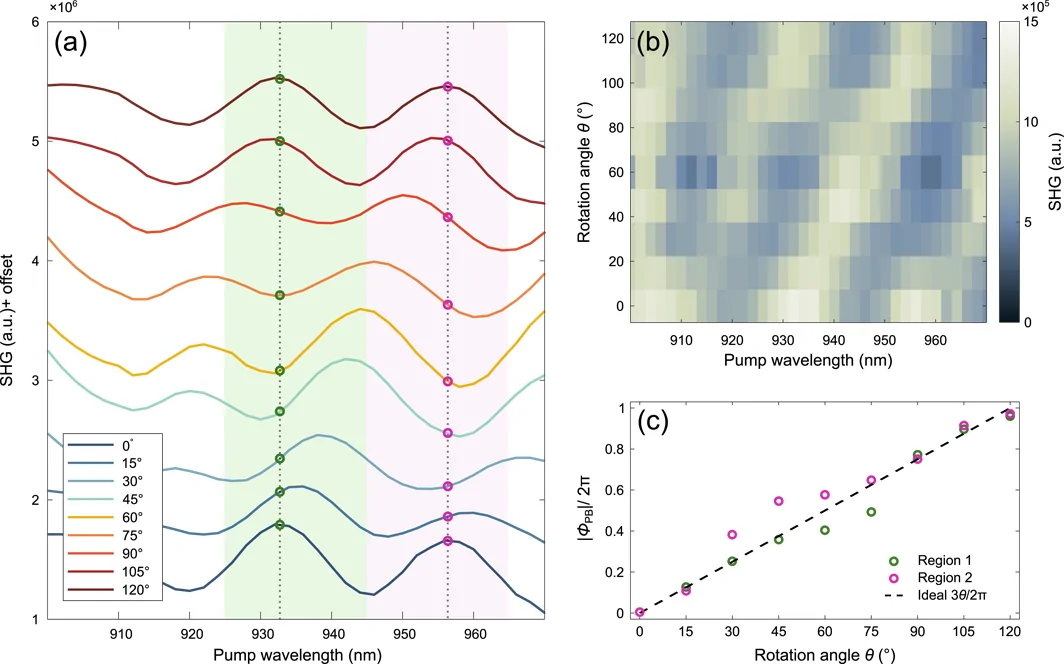
SHG measurements and geometric phase extraction. (a) SHG intensity spectra measured at different rotation angles θ. As the rotation angle increases, the phase‐matching peaks shift continuously across the spectrum. At θ=120°, each peak occupies the spectral position previously held by its neighbor, completing one full 2π geometric phase cycle. Spectra are vertically offset by $0.5\times 10^{6}$ a.u. for clarity. (b) Angle‐resolved SHG map showing the SHG intensity (color scale) as a function of wavelength (*x*‐axis) and rotation angle (*y*‐axis). The diagonal shift of the intensity maxima confirms the continuous spectral tuning of the phase‐matching peaks with rotation. (c) Geometric phase extracted from sinusoidal fits to the SHG intensity as a function of rotation angle, evaluated at the two representative wavelength regions highlighted in panel (a) (Peak 1, green circles; Peak 2, magenta circles). The extracted phase increases linearly with rotation angle, following the ideal dependence (dashed black line), and spans a full 2π range upon a 120° physical rotation, in good agreement with the theoretical prediction.

## 4 Conclusions and Outlook

In summary, we have demonstrated dynamic, mechanically reconfigurable control of SHG phase‐matching conditions using $C_{3v}$‐symmetric plasmonic metasurfaces integrated within an MPC. By introducing the nonlinear PB phase as a tunable bias in the phase‐matching condition, we showed that rotating one metasurface relative to the other continuously shifts discrete phase‐matching peaks across a 900–970 nm pump range. The threefold phase modification inherent to the $C_{3v}$ geometry enables a full 2π modulation with a 120° physical rotation, and the extracted geometric phase shows good agreement with the ideal 3σθ dependence across the full tuning range. Crucially, this reconfigurability is achieved without modifying the nanostructure geometry, material composition, or pump conditions.

The geometric phase control mechanism is decoupled from the SHG conversion efficiency itself, and could be combined with high‐efficiency nonlinear sources in hybrid platforms. For instance, pairing a conventional SHG source—such as an ultra‐thin slab of a nonlinear crystal—with a geometric‐phase metasurface could provide additional dispersion control via resonance‐based phase control [6, 45] or via the geometric phase as demonstrated here and in prior works [23, 30, 31, 46, 47]. Such hybrid schemes could provide a route toward nonlinear applications requiring broadband operation and higher‐order dispersion management [48, 49, 50], combining ultrathin nonlinear materials with the continuous, broadband, and post‐fabrication reconfigurability of the geometric phase.

A practical route toward increased efficiency would be to replace one or both mirrors of the MPC with a reflective metasurface—for instance, a resonant waveguide grating designed to simultaneously act as a high‐reflectivity mirror and a nonlinear geometric‐phase element [51, 52, 53, 54]—thereby achieving a more compact geometry with reduced propagation losses. More broadly, these results suggest that engineered rotational symmetry may serve as a useful design principle for reconfigurable nonlinear flat optics, opening new possibilities for tunable frequency conversion and nonlinear wavefront shaping in compact, ultrathin optical platforms.

## Author Contributions

M.M. and M.J.H. conceived the experiment. M.M. designed the metasurface samples, performed the experiments, analyzed the data, and wrote the first draft of the manuscript. R.C. fabricated the metasurface samples. T.K.H. supervised the fabrication. R.F. and M.J.H. supervised the work. All authors contributed to reviewing and revising the manuscript and approved the final version.

## Conflicts of Interest

The authors declare no conflicts of interest.

## Supporting information


Supporting Information S1

## Data Availability

The data that support the findings of this study are available from the corresponding author upon reasonable request.
