# Supplementary material for: Dynamic Control of Phase Matching in Nonlinear Metasurfaces Using Pancharatnam–Berry Phase
Source: Nanophotonics. 2026 Aug 28;15(17):e70273. doi: 10.1002/nap2.70273 (PMC13672128; doi:10.1002/nap2.70273)
Supplement: Supplementary file 1 [file NAP2-15-e70273-s001.pdf]

Active control of phase matching in nonlinear metasurfaces using Pancharatnam–Berry phase - Supplemental Material

Madona Mekhael[1], Roman Calpe[2], Tommi Hakala[2], Robert Fickler[1], and Mikko J. Huttunen[1,*]

[1]Photonics Laboratory, Physics Unit, Tampere University, Tampere, Finland
[2]Center for Photonics Sciences, University of Eastern Finland, Joensuu, Finland

Nonlinear Optical Measurements

The experimental setup used to characterize the second-harmonic generation (SHG) from the metasurfaces is described here in detail. A schematic of the setup is shown in Fig. 3 of the main text and reproduced in Fig. S1 for convenience. A tunable titanium:sapphire femtosecond laser (Coherent Chameleon Vision II), operating at a repetition rate of 80 MHz with a spectral range of 680–1080 nm and a pulse duration of around 200 fs, served as the pump source. The pump wavelength was scanned across the 900–970 nm range in steps of 2 nm at each rotation angle. A half-wave plate (HWP) combined with a linear polarizer (LP) was used to control the pump power incident on the sample. Three lenses L1 (f_1 = 75 mm), L2 (f_2 = 25.4 mm), and L3 (f_3 = 250 mm) were used to adjust the beam diameter and set the focusing conditions. A quarter-wave plate (QWP) placed after L3 converted the linearly polarized beam to right-circular polarization (RCP), and a long-pass filter (LPF) was used to suppress any residual wavelengths below the pump range before the beam entered the multipass cell.

The multipass cell (MPC) consisted of two high-reflectivity mirrors M1 and M2 (R $>$ 99%) enclosing two metasurface samples. The mirrors were aligned such that the pump beam interacted three times with each metasurface sample, establishing the phase-matching condition responsible for the peaks in the SHG spectrum. The first metasurface was mounted on a motorized rotation stage, allowing in-plane rotation by an angle θ_{yz} about the beam propagation axis (x). The beam waist at this metasurface was approximately 340 μm, corresponding to a Rayleigh range of 9.75 cm, ensuring uniform interaction with both metasurfaces throughout the multipass cell.

After the MPC, lens L4 (f_4 = 75 mm) collected and collimated the output beam. A dichroic mirror (DM) separated the SHG signal from the residual pump, directing the reflected pump beam to a camera for sample imaging and beam position control. The transmitted SHG signal was then focused by lens L5 (f_5 = 75 mm) and further filtered by a short-pass filter (SPF) to suppress any remaining pump light, before being imaged onto a sensitive CMOS camera (ZWO-ASI1600MM).

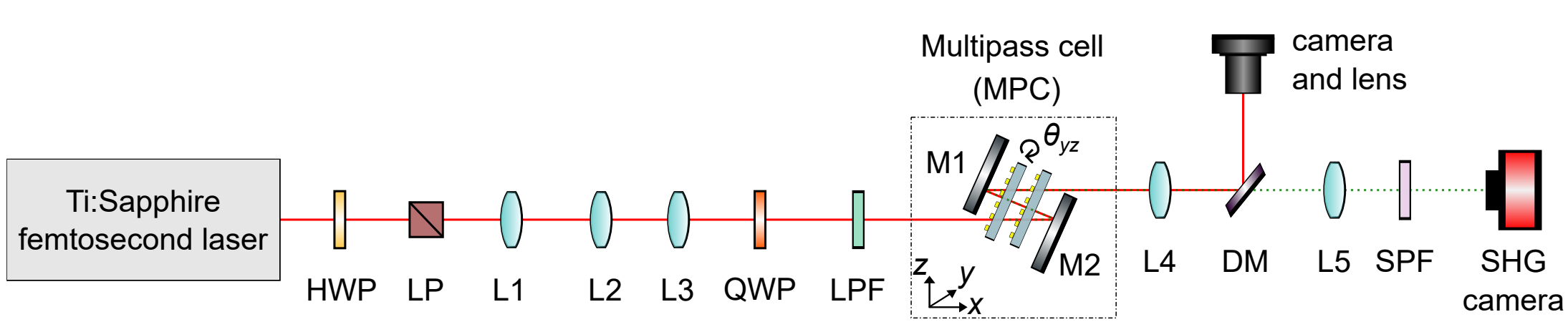

Figure S1: **Experimental setup.** Schematic of the setup used for SHG measurements from a dual-metasurface multipass cell at different rotation angles of one metasurface.
